# Gut phageome in Mexican Americans: a population at high risk for metabolic dysfunction-associated steatotic liver disease and diabetes

**DOI:** 10.1128/msystems.00434-24

**Published:** 2024-08-21

**Authors:** Suet-Ying Kwan, Caroline M. Sabotta, Lorenzo R. Cruz, Matthew C. Wong, Nadim J. Ajami, Joseph B. McCormick, Susan P. Fisher-Hoch, Laura Beretta

**Affiliations:** 1 Department of Molecular and Cellular Oncology, The University of Texas MD Anderson Cancer Center, Houston, Texas, USA; 2 The Platform for Innovative Microbiome and Translational Research (PRIME-TR), Moon Shots Program, The University of Texas MD Anderson Cancer Center, Houston, Texas, USA; 3 School of Public Health, University of Texas Health Science Center at Houston, Brownsville Regional Campus, Brownsville, Texas, USA; Kobenhavns Universitet, Frederiksberg C, Denmark

**Keywords:** metagenomics, healthcare disparities, gut phageome, liver steatosis, diabetes mellitus

## Abstract

Mexican Americans are disproportionally affected by metabolic dysfunction-associated steatotic liver disease (MASLD), which often co-occurs with diabetes. Despite extensive evidence on the causative role of the gut microbiome in MASLD, studies determining the involvement of the gut phageome are scarce. In this cross-sectional study, we characterized the gut phageome in Mexican Americans of South Texas by stool shotgun metagenomic sequencing of 340 subjects, concurrently screened for liver steatosis by transient elastography. Inter-individual variations in the phageome were associated with gender, country of birth, diabetes, and liver steatosis. The phage signatures for diabetes and liver steatosis were subsequently determined. Enrichment of *Inoviridae* was associated with both diabetes and liver steatosis. Diabetes was further associated with the enrichment of predominantly temperate *Escherichia* phages, some of which possessed virulence factors. Liver steatosis was associated with the depletion of *Lactococcus* phages r1t and BK5-T, and enrichment of the globally prevalent *Crassvirales* phages, including members of genus cluster IX (*Burzaovirus coli*, *Burzaovirus faecalis*) and VI (*Kahnovirus oralis*). The *Lactococcus* phages showed strong correlations and co-occurrence with *Lactococcus lactis*, while the *Crassvirales* phages, *B. coli*, *B. faecalis*, and UAG-readthrough crAss clade correlated and co-occurred with *Prevotella copri*. In conclusion, we identified the gut phageome signatures for two closely linked metabolic diseases with significant global burden. These phage signatures may have utility in risk modeling and disease prevention in this high-risk population, and identification of potential bacterial targets for phage therapy.

**IMPORTANCE:**

Phages influence human health and disease by shaping the gut bacterial community. Using stool samples from a high-risk Mexican American population, we provide insights into the gut phageome changes associated with diabetes and liver steatosis, two closely linked metabolic diseases with significant global burden. Common to both diseases was an enrichment of *Inoviridae*, a group of phages that infect bacterial hosts chronically without lysis, allowing them to significantly influence bacterial growth, virulence, motility, biofilm formation, and horizontal gene transfer. Diabetes was additionally associated with the enrichment of *Escherichia coli*-infecting phages, some of which contained virulence factors. Liver steatosis was additionally associated with the depletion of *Lactococcus lactis*-infecting phages, and enrichment of *Crassvirales* phages, a group of virulent phages with high global prevalence and persistence across generations. These phageome signatures may have utility in risk modeling, as well as identify potential bacterial targets for phage therapy.

## INTRODUCTION

Hispanics are the largest ethnic minority in the United States and a fast-growing population, with 19.1% of the total population in 2022 and a projected 26.9% by 2060 (1). Hispanics in the United States are disproportionately affected by chronic liver disease, which is the eighth-leading cause of mortality in this population with 2.6% of deaths (2). Hispanics are at the highest risk of metabolic dysfunction-associated steatotic liver disease (MASLD), which is often co-occurrent with obesity and diabetes (3). The increased risk of MASLD in Hispanic populations is nearly entirely attributable to Mexican Americans, with moderate and severe hepatic steatosis twice as common in Mexican Americans than European Americans (4).

Animal studies have demonstrated a causative role for the gut microbiome in MASLD development and progression. Indeed, absence of a gut microbiome in germ-free mice or antibiotic suppression of gut microbial growth, protect against diet-induced MASLD (5). Susceptibility to MASLD is also transmissible between mice following gut microbiota transplantation or cohousing (6–8).

Cross-sectional studies in humans identified specific bacterial taxonomic changes associated with MASLD development and progression (9–13). Most studies on the association between the gut microbiome and MASLD have focused on the bacterial microbiome. However, the gut is also home to other microorganisms, including a large population of viruses, of which over 90% are prokaryotic phages that infect bacteria (14). The adult human gut phageome contains a core set of persistant phages, which are temporally stable, yet highly heterogenous across individuals. Transiently detected phages are less abundant but shared across a larger percentage of individuals (15, 16). Phages influence human health and disease by shaping the gut bacterial community through either antagonistic or beneficial phage-bacterium interactions (17). Phages may also directly influence the human host through activation of the immune system, either locally or systemically (17). A recent study characterized the gut phageome in liver cirrhosis of different etiologies, identifying taxonomic and functional changes specific to each etiology (18). In this study, we aimed to identify alterations in the gut phageome associated with MASLD and diabetes in the high-risk population of Mexican Americans in South Texas. To that end, we enrolled subjects from the Cameron County Hispanic Cohort (CCHC), a large population-based Mexican American cohort in South Texas with high prevalence of obesity (51%), diabetes (28%), liver steatosis (52%), and liver fibrosis (14%) (19–22).

## RESULTS

### Phageome profiles identify subject clusters that differ by diabetes and liver steatosis

**Fig 1:**
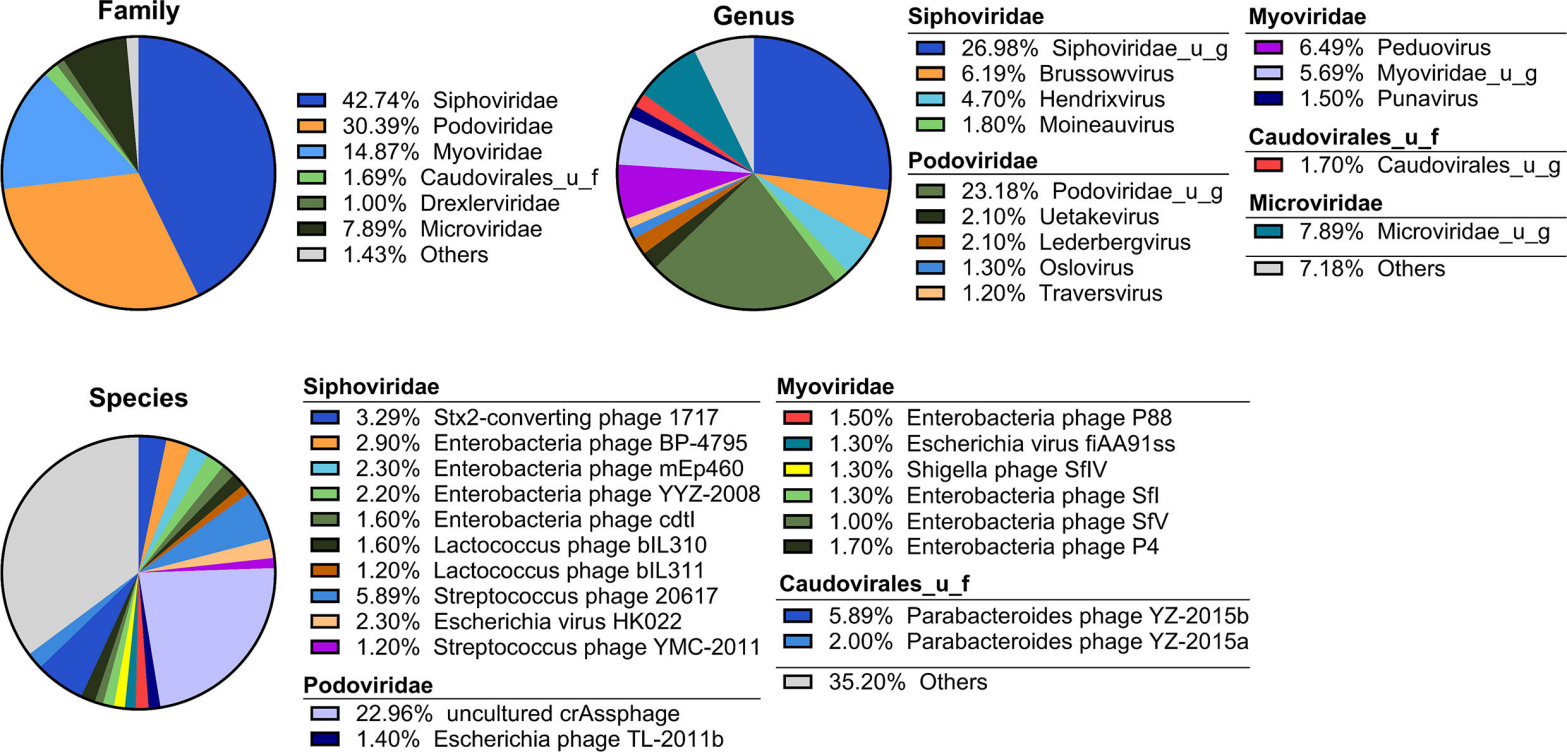
Abundance distribution of top phage families, genera, and species in the gut phageome of study participants. The mean percentage of reads classified as viral was 0.36% (range: <0.01%–17.07%). Pie charts were plotted using mean abundance across all subjects for those phage families, genera, and species with at least 5% incidence rate and at least 1% mean abundance in the 340 study participants. Others: taxa that did not pass the 1% mean abundance threshold.

We collected stool from 340 randomly selected subjects from the CCHC (Table S1). Among them, 239 (70.5%) were born in Mexico, 187 (55.2%) were obese and 124 (36.5%) were diabetic. Vibration-controlled transient elastography (VCTE) FibroScan screening showed that 229 (67.6%) had liver steatosis and 48 (14.1%) had significant liver fibrosis (kPa ≥7.1), including 29 (8.5%) with advanced fibrosis (kPa ≥8.8). Using shotgun metagenomic sequencing and taxonomic classification of all phages, we identified three orders, nine families, 32 genera, and 198 species detected in at least 5% of the samples (Table S2). The mean percentage of metagenomic reads classified as viral was 0.36% (range: <0.01%–17.07%). The mean number of viral species detected in each sample was 50 (range: 3–184). At the order level, the majority of reads were assigned to *Caudovirales* (91.6%), within which the most abundant families were *Siphoviridae* (42.7%), *Podoviridae* (30.4%), *Myoviridae* (14.9%), and unclassified *Caudovirales* (1.7%); and the *Petitvirales* order (7.9%), within which all reads were assigned to the *Microviridae* family (7.9%) (Fig. 1). The genera and species of these five phage families, with at least 1% mean abundance, are shown in Fig. 1.

**Fig 2:**
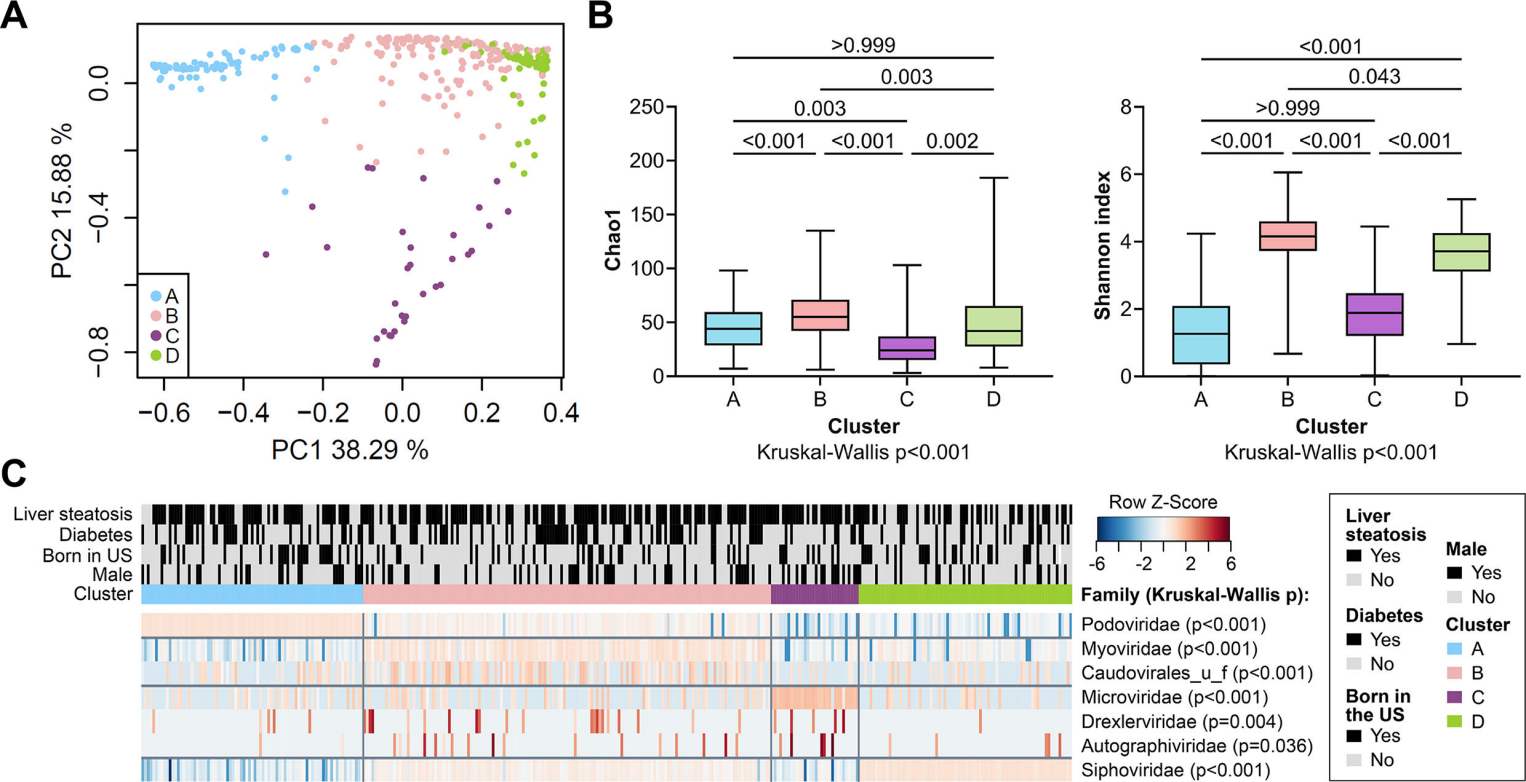
Samples clustering and contribution of demographic and clinical parameters and phage family to differences between clusters. (**A**) PCoA plot based on Brays-Curtis distances of phage family abundances. Samples were grouped by PAM clustering. (**B**) Alpha diversity, as measured by the Chao1 and Shannon index scores, in the different clusters. Bar charts show minimum to maximum, with quartiles. (**C**) Heatmap of phage families (log10 abundance) with significantly different abundance across clusters, as determined by Kruskal-Wallis test. Taxa are grouped depending on which cluster has the highest median abundance. Within each group, taxa are ordered by descending mean abundance. Color key represents row *Z*-scores. Distribution of demographic and clinical parameters significantly different among subjects in each cluster is shown.

Relationships between the gut phageome at the family level and demographic/clinical parameters were revealed by Partitioning Around Medoids clustering, resulting in four groups, with 81 subjects in cluster A, 149 in cluster B, 32 in cluster C, and 78 in cluster D (Fig. 2A). Alpha diversity by Chao1 or Shannon index, was significantly different between clusters (Fig. 2B). Cluster B had both the highest Chao1 index scores, measuring richness, and Shannon index scores, measuring richness and evenness. Clusters C and A had the lowest Chao1 scores and Shannon scores, respectively. The demographic and clinical characteristics associated with each cluster were examined by logistic regression analysis (Table S3; Fig. 2C). Abundance of specific families significantly enriched or depleted in each cluster, was assessed by the Kruskal-Wallis test (Fig. 2C). No significant difference in the parameters tested was observed in Cluster A subjects, characterized by high abundance of *Podoviridae*. Subjects in cluster B, characterized by high abundance of *Myoviridae* and unclassified *Caudovirales*, were least likely to have been born in the United States [21.5% vs 32.6%; odds ratio (OR) = 0.58; 95% confidence interval (CI) = 0.35–0.95, *P* = 0.029], Cluster B had also the highest frequency of subjects with diabetes (43.8% vs 31.6%; OR = 1.69; 95% CI = 1.08–2.65, *P* = 0.021). Cluster C, characterized by high abundance of *Microviridae*, *Drexlerviridae*, and *Autographiviridae*, had the highest percentage of males (46.9% vs 28.2%; OR = 2.24; 95% CI = 1.07–4.69, *P* = 0.032) and frequency of elevated AST levels (18.8% vs 7.5%; OR = 2.83; 95% CI = 1.06–7.57, *P* = 0.038). Finally, cluster D, characterized by high abundance of *Siphoviridae*, had the lowest frequency of liver steatosis (53.8% vs 71.6%; OR = 0.46; 95% CI = 0.27–0.78, *P* = 0.004). Overall, these results suggested that inter-individual variations in the gut phageome in the study participants, were associated with gender, country of birth, liver steatosis, and diabetes.

### Gut phageome signatures associated with diabetes

**Fig 3:**
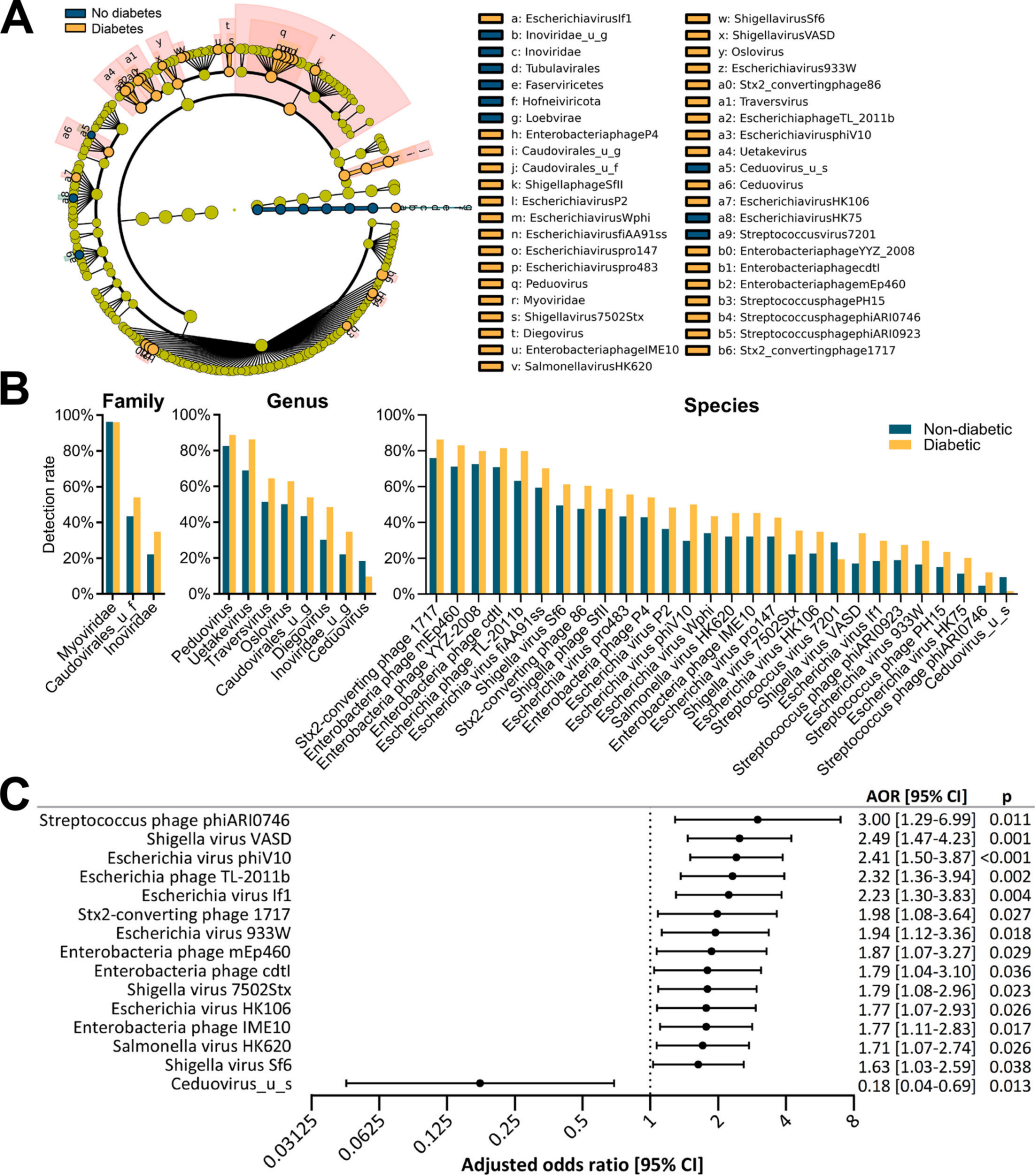
Phages with altered abundance in subjects with diabetes. (**A**) Cladogram showing taxa detected in at least 5% of samples with significantly different relative abundance between subjects with and without diabetes, as assessed by the linear discriminant analysis (LDA) effect size (LEfSe) algorithm. Significance was determined using LEfSe LDA >2.0. (**B**) Detection rates in subjects with diabetes and in subjects without diabetes, of family, genera, and species from panel (A), which also remained significant by ANCOM (FDR <0.2). (**C**) Forest plot of significant species from panel (B), which showed a significant association with diabetes after adjusting for age and gender. Adjusted odds ratios (AORs) for diabetes are shown for subjects with presence of the phage species.

To identify phages enriched or depleted in subjects with diabetes, linear discriminant analysis (LDA) effect size (LEfSe) analysis of taxa was performed. A total of 43 taxa were identified, all remaining significant by ANCOM analysis (FDR <0.2) (Fig. 3A). Figure 3B shows the difference in detection rates between patients with and patients without diabetes, from families to species. Logistic regression analysis, adjusting for age and gender, further confirmed the association between phage presence and diabetes for 15 of the 28 species identified (Fig. 3C). The strongest positive associations with diabetes were observed for *Streptococcus* phage phiARI0746 [adjusted odds ratio (AOR) = 3.00; 95% CI = 1.29–6.99, *P* = 0.011], *Shigella* virus VASD (AOR = 2.49; 95% CI = 1.47–4.23, *P* = 0.001)*, Escherichia* virus phiV10 (AOR = 2.41; 95% CI = 1.50–3.87, *P* < 0.001), and *Escherichia* phage TL-2011b (AOR = 2.32; 95% CI = 1.36–3.94, *P* = 0.002). The only negative association with diabetes was observed for *Ceduovirus_u_s* (AOR = 0.18; 95% CI = 0.04–0.69, *P* = 0.013). All phages were predicted to be temperate, with the exception of *Escherichia* virus If1 (Table S4). Five of the phages positively associated with diabetes (*Shigella* virus VASD, *Shigella* virus 7502Stx, Stx2-converting phage 1717, *Escherichia* virus 933W, and *Enterobacteria* phage cdtl) were predicted to possess virulent factors (Table S4).

### Gut phageome signatures associated with liver steatosis

**Fig 4:**
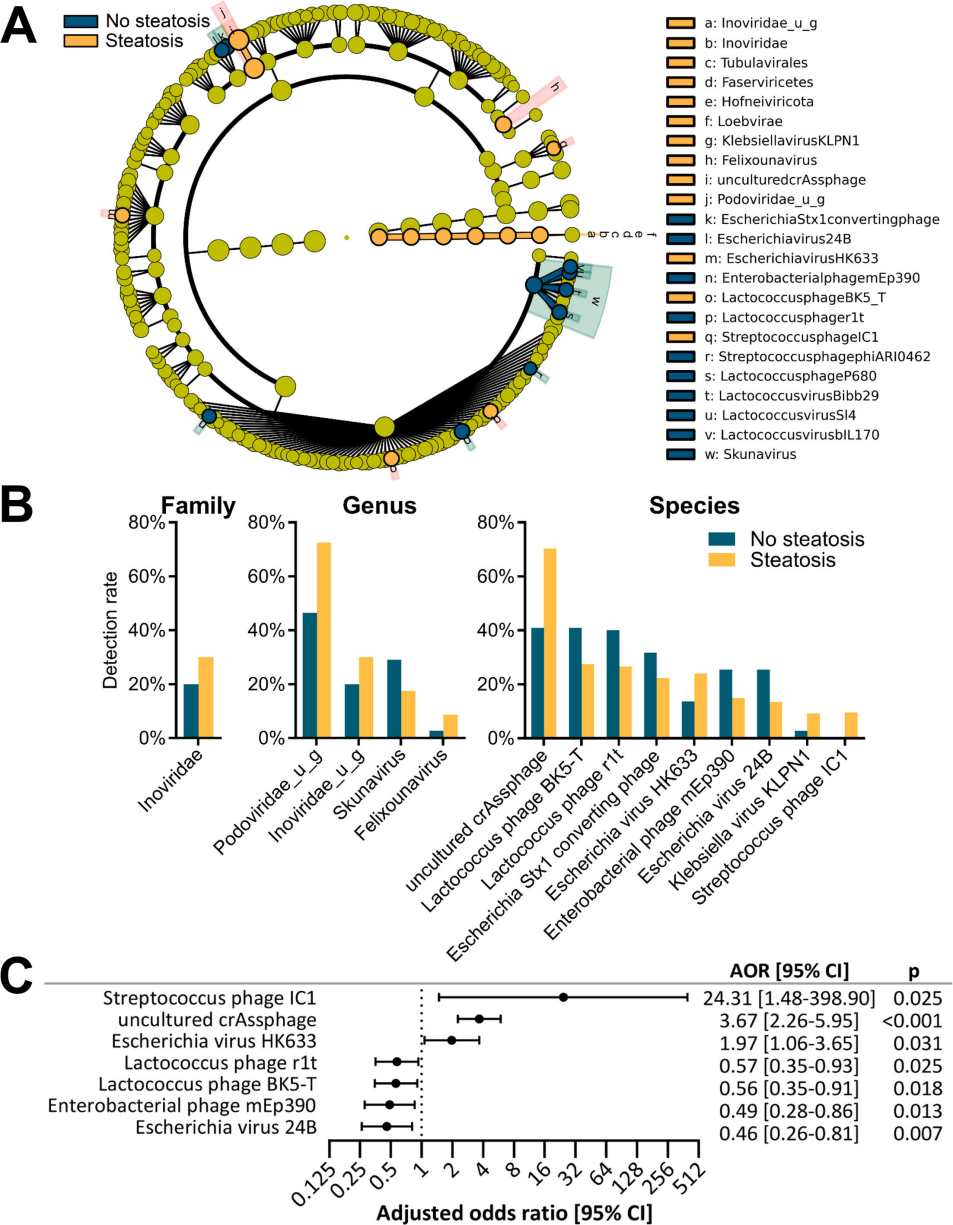
Phages with altered abundance in subjects with steatosis. (**A**) Cladogram showing taxa detected in at least 5% of samples with significantly different relative abundance between subjects with and without steatosis, as assessed by the linear discriminant analysis (LDA) effect size (LEfSe) algorithm. Significance was determined using LEfSe LDA >2.0. (**B**) Detection rates in subjects with steatosis and in subjects without steatosis, of family, genera, and species from panel (A), which also remained significant by ANCOM (FDR <0.2). (**C**) Forest plot of significant species from panel (B), which showed a significant association with liver steatosis after adjusting for age and gender. Adjusted odds ratios (AORs) for liver steatosis are shown for subjects with presence of the phage species.

We also identified by LEfSe analysis, 23 taxa enriched or depleted in subjects with liver steatosis (Fig. 4A). Of these, 18 remained significant by ANCOM analysis (FDR <2.0), with Fig. 4B showing the difference in detection rates between patients with and patients without steatosis from families to species. A strong detection rate increase with steatosis was observed for the genus *Podoviridae_u_g* (46.4% vs 72.5%) while a strong detection rate decrease was observed for the genus *Skunavirus* (29.1% vs 17.5%). Interestingly, increased detection of the *Inoviridae* family was common to both subjects with diabetes and subjects with steatosis. For seven of the nine species significant by LEfSe and ANCOM, logistic regression analysis, adjusting for age and gender, further confirmed that their presence was significantly associated with steatosis (Fig. 4C). Strong positive associations were observed for *Streptococcus* phage IC1 (AOR = 24.31; 95% CI = 1.48–398.90, *P* = 0.025), uncultured *crAssphage* (AOR = 3.67; 95% CI = 2.26–5.95; *P* < 0.001), and *Escherichia* virus HK633 (AOR = 1.97; 95% CI = 1.06–3.65; *P* = 0.031). Negative associations were observed for *Escherichia* virus 24B (AOR = 0.46; 95% CI = 0.26–0.81, *P* = 0.007), *Enterobacterial* phage mEp390 (AOR = 0.49; 95% CI = 0.28–0.86, *P* = 0.013), and two *Lactococcus* phages r1T and BK5-T (AOR = 0.57; 95% CI = 0.35–0.93, *P* = 0.025 and AOR = 0.56; 95% CI = 0.35–0.91, *P* = 0.018, respectively). All phages were predicted to be temperate, while *Streptococcus* phage IC1 was predicted to possess one virulent factor (Table S4).

### crAssphages and liver steatosis

**Fig 5:**
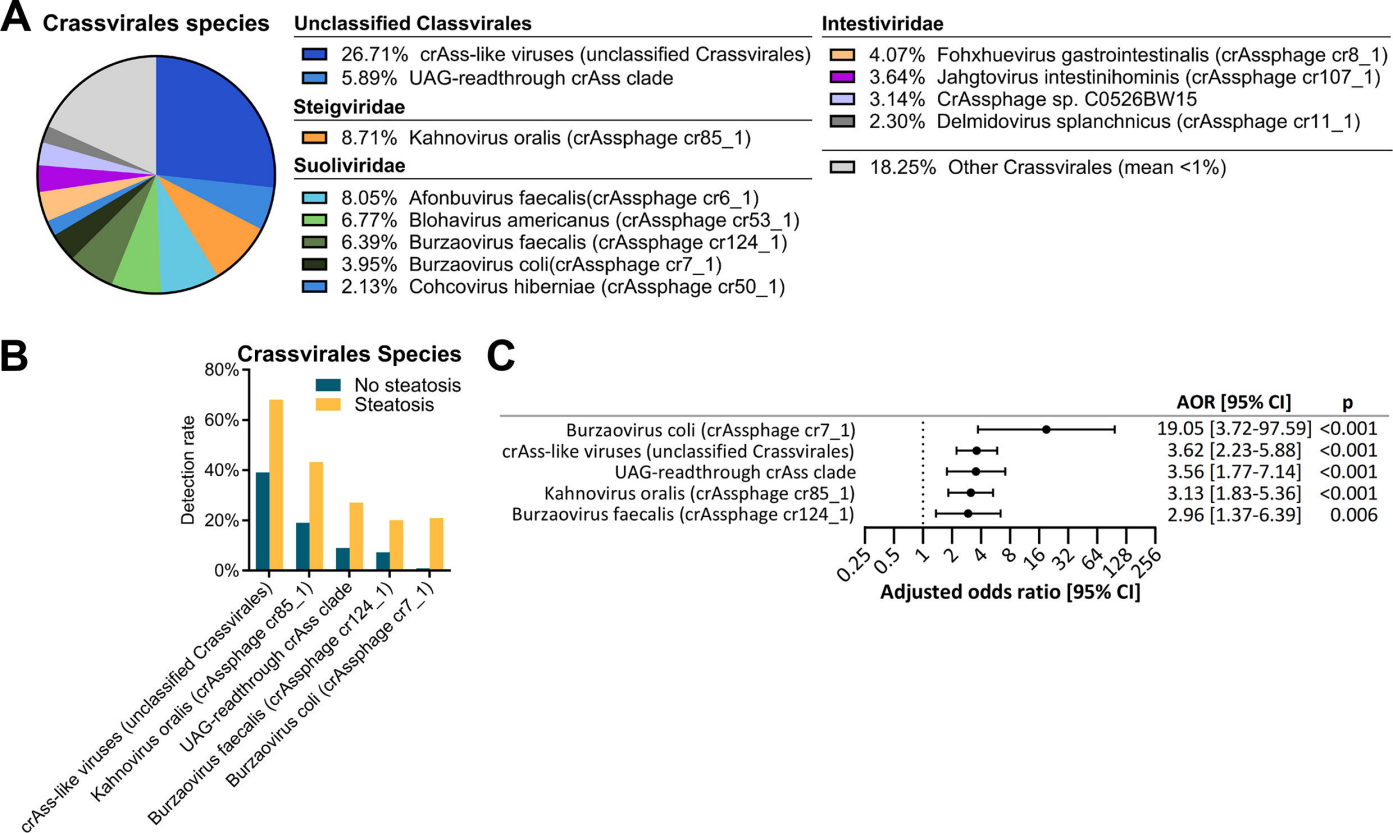
*Crassvirales* species with altered abundance in subjects with steatosis. The mean percentage of viral reads classified as *Crassvirales* was 47.7% (range 0.0%–100.0%), while the mean percentage of all reads classified as *Crassvirales* was 0.29% (range 0.00%–17.06%). (**A**) Abundance distribution of top *Crassvirales* phage species, with at least 5% incidence rate in the 340 study participants. Others: *Crassvirales* phage species with less than 1% mean abundance threshold. (**B**) Detection rates of Crassvirales phages with significant abundance differences between subjects with and subjects without liver steatosis. (**C**) Forest plot of species from panel (B), all showing a significant association with liver steatosis after adjusting for age and gender. Adjusted odds ratios (AORs) for liver steatosis are shown for subjects with presence of the phage species.

Uncultured crAssphages were originally assigned to the Podoviridae family, but have recently been re-assigned to a newly-formed *Crassvirales* order (23). Shotgun metagenomic sequencing reads were additionally processed through the VirMAP pipeline in an attempt to identify specific members of *Crassvirales*. A total of 47.7% of all mapped reads aligned to the *Crassvirales* order, of which 67.4% were successfully assigned to a named species. *Crassvirales* were detected in 97.6% of the 340 subjects, with 25 *Crassvirales* phage species detected in at least 5% of all samples (Table S5). The most abundant species were unclassified *Crassvirales* (12.7%), *Kahnovirus oralis* (4.2%), *Afonbuvirus faecalis* (3.8%), *Blohavirus americanus* (3.2%), and *Burzaovirus faecalis* (3.0%) (Fig. 5A). Among the 25 *Crassvirales* phage species, five taxa had detection rate increases in subjects with liver steatosis, and their presence were associated with increased risk of liver steatosis by logistic regression: *Burzaovirus coli* (21.0% vs 0.9%; AOR = 19.05; 95% CI = 3.72–97.59, *P* < 0.001), *B. faecalis* (20.1% vs 7.3%; AOR = 2.96; 95% CI = 1.37–6.39, *P* = 0.006), *K. oralis* (43.2% vs 19.1%; AOR = 3.13; 95% CI = 1.83–5.36, *P* < 0.001), the unnamed UAG-readthrough crass clade (27.1% vs 9.1%; AOR = 3.56 [95% CI = 1.77-7.14], *P* < 0.001), and unclassified *Crassvirales* (68.1% vs 39.1%; AOR = 3.62; 95% CI = 2.23–5.88, *P* < 0.001) (Fig. 5B and C). All three named *Crassvirales* phage species were predicted to be virulent; however, no virulent factors were identified (Table S4).

### Correlation analysis of steatosis- and diabetes-associated phage species with bacterial species

**Fig 6:**
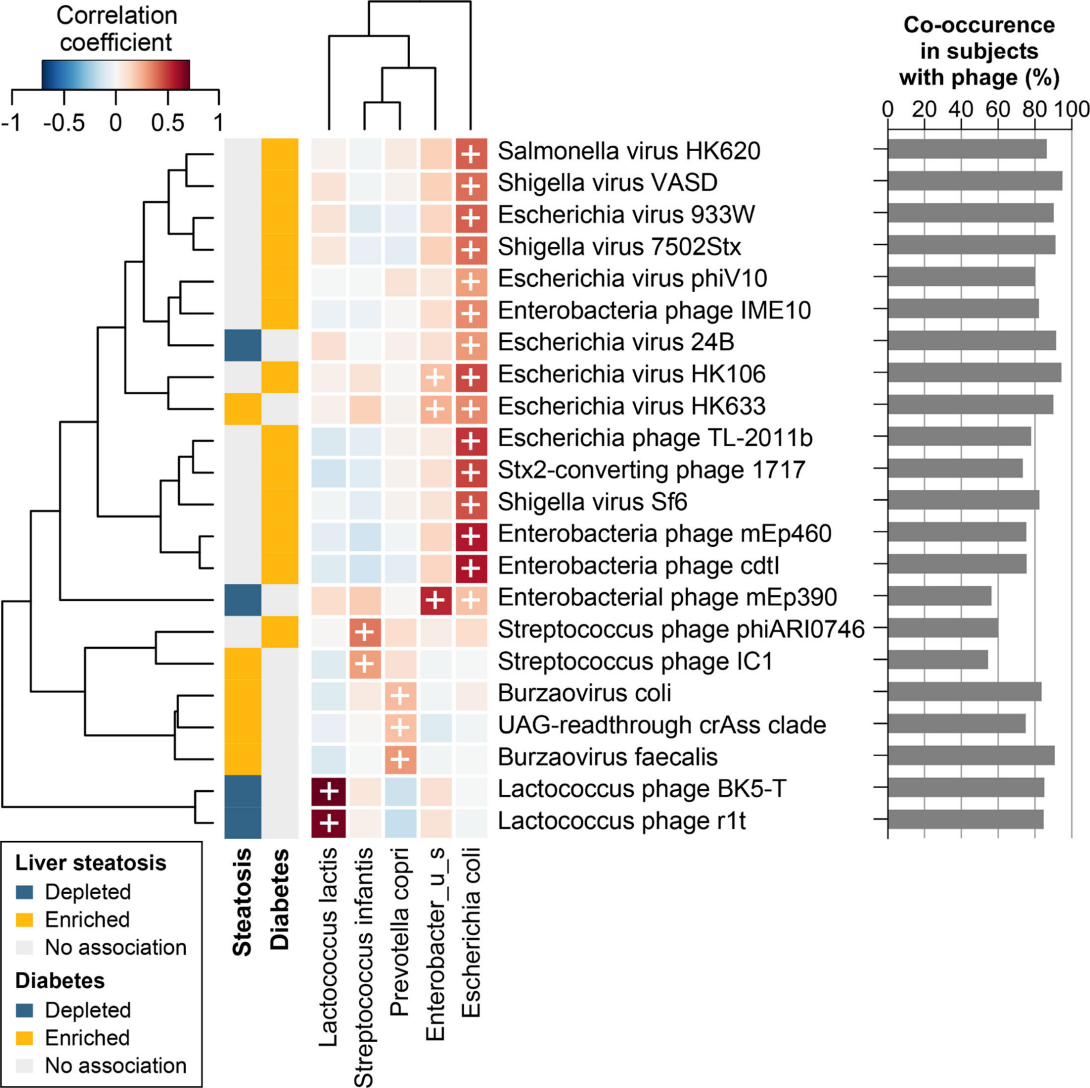
Co-occurrence analysis of disease-associated phages and potential bacterial host species. Left, spearman’s correlation matrix of relative abundances, between steatosis- and diabetes-associated phages with bacterial species. Bacterial species with the strongest correlation with each phage are shown. Right, percentage of samples in which the indicated phage and bacterial species pairs co-occur. Percentages were calculated among subjects where the phage was detected.

We next aimed to identify potential bacterial hosts of all diabetes- and steatosis-associated phage species identified. To that end, we selected the bacterial species with the strongest correlation to each phage (*r*_s_ ≥0.20) and a co-occurrence rate of ≥50% in subjects with presence of the phage (Fig. 6). Of the 14 phage species positively associated with diabetes, 12 strongly correlated with *Escherichia coli* (*r*_s_ = 0.29–0.56, *P* < 0.001), with co-occurrence ranging from 73.4% to 94.9% in subjects with presence of the phage (Fig. 6). These results were in agreement with annotation data on known or inferred hosts from PhageScope, with *E. coli* being the bacterial host for 9 of the 12 phages species. The other three phage species were predicted to infect the closely related *Shigella* species of the same family (Table S4). Importantly, both the detection rate and relative abundance of *E. coli* were greater in subjects with diabetes (detection rate: 75.0% vs 56.0%, *P* = 0.001; relative abundance: 0.03% vs 0.01%, *P* = 0.003). *Streptococcus* phage phiARI0746 strongly correlated with *Streptococcus infantis* (*r*_s_ = 0.37, *P* < 0.001; 60% co-occurrence) (Fig. 6). This phage was predicted to infect the closely related *Steptococcus pneumoniae* of the same genus (Table S4).

For the steatosis-associated phages, *Escherichia* viruses 24B and HK633 showed strong correlation and high co-occurrence also with *E. coli* (*r*_s_ = 0.30, *P* < 0.001; 91.5% co-occurrence and *r*_s_ = 0.33, *P* < 0.001; 90.0% co-occurrence, respectively), while *Lactococcus* phages r1t and BK5-T showed the strongest correlation and high co-occurrence with *Lactococcus lactis* (*r*_s_ = 0.67, *P* < 0.001, 84.8% co-occurrence and *r*_s_ = 0.70, *P* < 0.001, 85.2% co-occurrence, respectively) (Fig. 6). All four of these bacterial hosts were in concordance with predicted phage-host pairings in PhageScope (Table S4). The detection rate and relative abundance of *L. lactis* were also significantly lower in subjects with steatosis (detection rate: 36.2% vs 52.7%, *P* = 0.005; relative abundance: 0.00% vs 0.01%, *P* = 0.026). While no predicted bacterial host information was available in PhageScope for the *Crassvirales* phages associated with steatosis, *B. coli*, *B. faecalis*, and UAG-readthrough crAss clade showed a positive correlation and high co-occurrence with *Prevotella copri* (*r*_s_ = 0.22, *P* < 0.001, 83.7% co-occurrence; *r*_s_ = 0.30, *P* < 0.001, 90.7% co-occurrence; *r*_s_ = 0.20, *P* < 0.001, 75.0% co-occurrence, respectively) (Fig. 6). The detection rate and relative abundance of *P. copri* was significantly greater in subjects with steatosis (detection rate: 63.8% vs 36.4%, *P* < 0.001; relative abundance: 0.02% vs 0.00%, *P* = 0.002). Despite the increased detection of *P. copri* in subjects with liver steatosis, the increased detection of the *P. copri*-associated phages was of much greater magnitude. Consequently, steatotic subjects with *P. copri* were significantly more likely to contain *Crassvirales* members *Burzovirus coli* and UAG-readthrough crass clade than non-steatotic subjects with *P. copri* (Fig. S1).

## DISCUSSION

In light of the increasing global burden of antimicrobial-resistant bacteria, there has been a renewed interest in the therapeutic potential of phages in targeting specific bacterial species, particularly for infectious diseases. Due to the involvement of the bacterial gut microbiome in MASLD, interest has also extended to the treatment of MASLD (14). Preclinical studies demonstrated an attenuation of MASLD-related steatohepatitis after administration of phages targeting *Klebsiella pneumoniae* (24, 25). However, studies on the gut phageome in MASLD are scarce (18, 26). In this study, we aimed to characterize the gut phageome of Mexican Americans in South Texas, with high incidences of MASLD and metabolic comorbidities.

Three hundred forty study participants from CCHC were separated into four main clusters based on their gut phageome profiles, with country of birth differing between them. This is concordant with the observation that the gut phageome exhibits global variation, with geographical location being a major factor influencing the gut phageome (27, 28). The infant gut is rapidly colonized by a diverse range of phages originating from maternal, dietary, and environmental sources, and stabilizes by adulthood (29). Incidence of diabetes and liver steatosis also differed between the four clusters. It remains to be determined whether the phageome clusters we observed persist across populations. While gut bacterial enterotypes are well-known to persist across populations, studies reporting on phage enterotypes are limited. In patients with IBD, Jansen et al. observed two viral clusters distinguished by high and low crAssphage abundance (30). Conversely, Camarillo-Guerrero et al. reported that samples clustered by lifestyle (rural vs urban), with the rural and urban clusters associating with *Prevotellaceae* and *Bacteroides* as the bacterial host, respectively (28). Due to the bacterial host specificity of phages and the finding that phage clusters were associated with the *Prevotellaceae*-enriched and *Bacteroides*-enriched bacterial enterotypes, it is possible that phage enterotypes may also persist across populations.

The *Inoviridae* family was the only taxon associated with both diabetes and liver steatosis, with *Escherichia* virus If1 as the most abundant species. Detected in 26.8% of subjects, the incidence of liver steatosis was 75.8% for subjects with detected *Inoviridae* compared to 63.3%, and the incidence of diabetes was 47.3% for subjects with detected *Inoviridae* compared to 32.5%. An enrichment of *Inoviridae* has been reported in patients with MASLD-related liver cirrhosis compared to cirrhosis of other etiologies (18). *Inoviridae* was also recently found to positively correlate with proinflammatory cytokines in children with type-2 diabetes (31). Currently characterized members of this family were found to represent only a small fraction of a highly diverse group of phages that infect a phylogenetically broad range of bacterial hosts (32). *Inoviridae* is a family of non-enveloped bacteriophages characterized by filamentous morphology and a chronic infection cycle (17, 32). In contrast to virulent phages (replicating strictly by a lytic cycle) and temperate phages (replicating by both lysogenic and lytic cycles), filamentous phages undergoing a chronic cycle continuously produce and release virions without lysis of the host bacterial cell. The absence of a lytic cycle allows *Inoviridae* to significantly influence host cell growth, motility, biofilm formation, virulence, and horizontal gene transfer, thus providing a potential mechanism by which phages affect human host phenotype indirectly through modulation of the gut bacterial microbiome (33, 34).

According to phage lifestyle prediction from PhageScope, apart from *Escherichia* phage If1, all other phages positively associated with diabetes were temperate phages of the *Podoviridae* and *Siphoviridae* families, with the majority known to infect *E. coli*, and displaying strong correlations with abundance of *E. coli*. Accordingly, detection rates and relative abundance of *E. coli* were also significantly increased in subjects with diabetes. We previously reported that *Escherichia* was enriched in CCHC subjects with diabetes in a smaller sample set (35). *Escherichia* phages were also reported to be associated with increased blood glucose in patients with MASLD (26). Several of the diabetes-associated *Shigella* and *Escherichia* phages identified, were predicted to possess virulent factors, most notably Shiga toxin genes 1 and 2 (Stx1 and Stx2). Shiga toxins, composed of subunits A and B, are the major virulence factors of shiga toxin-producing *E. coli*, a subset of *E. coli* strains that are pathogenic and capable of causing a range of diseases. Shiga toxin genes are absent from *E. coli* chromosomes, and are instead provided by the presence of at least one prophage in the bacterial cell. Temperate phages are capable of both lysogenic and lytic cycles. In the lysogenic cycle, phages replicate without virion production or bacterial lysis. A switch from the lysogenic cycle to the lytic cycle occurs spontaneously or due to external stressors to the bacterial host, including antibiotic treatment (36). The majority of phage genes, including Stx, remain dormant during the lysogenic phase, but are induced during the switch to a lytic cycle (37). Clinical studies have tested phages targeting pathogenic *E. coli* for the treatment of gastrointestinal diseases (14). In a preclinical model, a temperate phage genetically engineered to deliver a transcriptional repressor of Stx, reduced fecal Stx concentrations (38). Our findings suggest that phages targeting *E. coli* and its virulence factors, may also have clinical utility in diabetes.

Liver steatosis was similarly associated with changes in several temperate phages, including a negative association with *Lactococcus* phages r1t and BK5-T, concurrent with the depletion of their putative host, *L. lactis*. Conversely, *Escherichia* virus HK633 was positively associated with liver steatosis. A previous study of MASLD patients found that *Lactococcus* phage BK5-T was negatively correlated with BMI, while *Escherichia* virus HK633 was positively correlated with blood glucose levels (26). *Streptoccocus* phage IC1 displayed the strongest positive association with liver steatosis. This was the only steatosis-associated phage predicted to possess virulence factors, namely lytic amidase. Phage-encoded lytic amidase is orthologous to the bacteria-encoded virulence factor, autolysin (LytA) (39). It remains to be seen how phage-encoded lytic amidase may affect the virulence of its host, given the functional redundancies.

In addition to temperate phages, subjects with liver steatosis were also found to have increased prevalence of several phages in the dominant, virulent group of *Crassvirales* phages. The crAss-like phages were originally observed to have podovirus-like morphology and assigned to the *Podoviridae* family (17), but were recently formed into a new *Crassvirales* order, comprised of four families, 11 subfamilies, 42 genera, and 73 species (23). The high prevalence of *Crassvirales* in the CCHC subjects, is in agreement with the literature, where crAss-like phages were found to be the most abundant phage species, detected globally in over half of all gut metagenomes (28). Despite being strictly lytic, crAss-like phages are temporally stable and can persist across generations (40). Uncultured crAssphages were the most prevalent phage in patients with liver cirrhosis, and higher in patients of MASLD etiology than in alcohol-induced cirrhosis (18). However, no named crAssphages were identified by the taxonomic classification pipeline used in that study. Using VirMap, a separate pipeline for taxonomic classification, we confirmed a strong positive association between several *Crassvirales* phages and liver steatosis. *B. coli* displayed a particularly strong positive association with steatosis. Detected in 14.4% of all subjects, the incidence of liver steatosis was 98.0% for subjects with detected *B. coli* (compared to 62.4%). Among the three named *Crassvirales* phage species positively associated with liver steatosis, *B. coli* and *B. faecalis* belong to the *Suoliviridae* family (previously group Delta) and *Oafivirinae* subfamily (previously candidate genus IX), while *K. oralis* belongs to the *Steigviridae* family (previously group Beta) and *Asinivirinae* subfamily (previously candidate genus VI). Gut samples from Westernized, industrialized regions were previously observed to be enriched in genus I, while genus VI and IX were more prevalent in rural, non-Westernized regions (28, 41). Both of the *Burzaovirus* phages and UAG-readthrough crAss clade showed strong correlation and high co-occurrence with *P. copri*, a member of the *Bacteroidetes* phylum. This is in accordance with reports of *Bacteroidetes* being the major host of crAss-like phages, and with *P. copri* being the putative host of IX crAssphages (28) and some Delta group crAssphages (42). We previously reported that *P. copri* was enriched in subjects with MASLD-related liver fibrosis ()(10). Interestingly, although detection rates and relative abundance of *P. copri* were also significantly increased in subjects with liver steatosis, steatotic subjects with *P. copri* were significantly more likely to contain *Crassvirales* members *Burzovirus coli* and UAG-readthrough crass clade than non-steatotic subjects with *P. copri*. It is possible that the presence of these *Crassvirales* phages modulates the behavior of their putative bacterial host, in a way that promotes liver steatosis.

This study applied metagenomic sequencing to bulk fecal samples, without prior enrichment of virus-like particles (VLPs). The average number of viral species detected in each sample was 50 (range 3–184). The main determinant of viruses detection was found to be sequencing depth, rather than source of sample (bulk vs VLP enriched) (28). Because fecal samples contain low percentages of viral sequences, such approach may lead to low sensitivity for detection of rare phages. The enrichment of VLP can also result in several limitations such as susceptibility to RNA degradation, biases in virus genome fragmentation, skewed abundances of certain viruses, and high variability between study protocols (43, 44). Remarkably, while the number of viral contigs detected in human fecal samples does not differ significantly between the two methods, the overlap in viruses detected by both methods is low (45). Bulk metagenomes are more likely to retain temperate phages that have integrated into the bacterial host genome as prophages, while VLP enriches for non-integrative phages, ssDNA phages, and RNA phages (43, 45).

In conclusion, Mexican Americans with diabetes display an enrichment of multiple *E. coli* phages, of which the majority were temperate, and some of which possessed virulence factors. Liver steatosis was associated with the depletion of *L. lactis*-infecting phages and enrichment of *Crassvirales* phages of the IX and VI genus clusters, with the IX phages predicted to infect *P. copri*. An enrichment of the filamentous *Inoviridae* was associated with both diseases. Additional studies would provide further insight on how these phages shape the gut bacterial community and contribute to metabolic disease. Furthermore, while virulent phages have been the main focus for phage therapy, temperate phages could also be exploited for therapeutic purposes, by engineering them to become exclusively lytic, or to deliver key genes (46). These phageome signatures may also have utility in risk modeling in this high-risk population.

## MATERIALS AND METHODS

### Study participants

This cross-sectional study includes 340 participants from the CCHC, a population-based cohort of Mexican Americans randomly recruited from households in Brownsville, Texas (19). The participants included were those with clinical visits and stool collected between February 2018 and August 2021. We excluded subjects positive for hepatitis B or C virus or who had antibiotic, probiotic, or proton pump inhibitor use within 30 days of stool collection. Written informed consent was obtained from each participant, and the study protocol was approved by the Institutional Review Board of the University of Texas MD Anderson Cancer Center (approved protocol number PA13-0317). At clinical visit, fasting blood samples were collected and analyzed for metabolic and lipid panels. The following criteria were used as categorical or diagnostic definitions: obesity (BMI ≥30), pre-diabetes (no history of diabetic medication, plus fasting blood glucose of 100–125 mg/dL or HbA1c of 5.7%–6.4%), diabetes (fasting blood glucose ≥126 mg/dL, HbA1c ≥6.5% or history of diabetic medication), elevated aspartate aminotransferase (AST) (>33 U/L), elevated alanine aminotransferase (ALT) (>40 U/L for men, >31 U/L for women), heavy drinking (alcohol consumption of >20 g/day for men, >10 g/day for women), moderate drinking (non-zero weekly consumption that did not reach the criteria for heavy drinking), former smoking (lifetime consumption of ≥100 cigarettes plus no smoking at time of survey), and current smoking (lifetime consumption of ≥100 cigarettes plus smoking at time of survey). Demographic and laboratory parameters of the study participants are described in Table S1.

All subjects were screened with VCTE (FibroScan 502 Touch or FibroScan 530 Compact, Echosens). Trained operators obtained controlled attenuation parameter (CAP) measurements (dB/m) for liver steatosis and liver stiffness measurements (LSM, kiloPascals, kPa) for liver fibrosis. Presence of liver steatosis was defined as CAP ≥268, as described (47). Significant liver fibrosis (F2–F4) was defined as LSM ≥7.1 kPa, while advanced fibrosis (F3–F4) was defined as LSM ≥8.8 kPa, as described (48). LSM measurements were considered inconclusive if <10 valid measures or interquartile range-to-median ratio >0.3.

### Stool DNA extraction, shotgun metagenomic sequencing, and bioinformatic analysis

Stool samples were collected from study participants using the OMNIgene GUT stool collection kit (DNA Genotek, Ontario, Canada), received within a median of 4 days from sample collection, aliquoted, and frozen at −80°C until use. The median time interval between clinical visit and stool collection was 5 days. Shotgun metagenomic sequencing was performed (CosmosID Inc., Rockville, Maryland) to a depth of 12 million reads (±20%). Samples were sequenced in three batches, with clinical outcomes randomized among batches to avoid confounding effects. DNA was isolated using the QIAGEN Dneasy PowerSoil Pro Kit (Qiagen) and quantified by Qubit (ThermoFisher). DNA libraries were prepared using the Illumina Nextera XT library preparation kit. Libraries were assessed with Qubit (ThermoFisher) and sequenced on an Illumina platform using 150 bp paired-end sequencing. The percentage of sequencing reads aligned to the human genome was determined to be minimal (mean of 0.04%) via Bowtie2 (v2.4.1) (49), using GRCh38 and major single nucleotide polymorphisms (SNPs) as the reference genome, and default Bowtie2 parameters. Unassembled sequencing reads were directly analyzed by the CosmosID bioinformatics platform, which utilizes a high-performance data-mining K-mer-based algorithm and curated genomic databases (50). Abundance scores, relative abundances, and the alpha diversity metrics, Chao1 index, and Shannon index, were used for downstream analysis. For further resolution within the group of crAss-like phages, the raw sequencing data were processed again through the VirMAP pipeline, which classifies the taxonomy of sequences by a combination of nucleotide and amino acid information (51). Alignment with the VirMAP pipeline was performed using the 2021 version of the Genbank viral (gbvrl) and phage (gbphg) divisions. All reads assigned to crAss-like phage species of the recently formed order of *Crassvirales* (23) were further analyzed. For phage annotation, information on host range, predicted phage lifestyle (temperate vs virulent), and presence of virulence factor genes, was retrieved from PhageScope (52), using the RefSeq ID of each phage. For the lifestyle annotation in PhageScope, phages were considered temperate if previously identified as such by mining using a computational temperate phage detection method, TemPhD (53). For the remaining phages, lifestyle prediction was performed using the Graphage tool (54).

### Statistical analyses

Unless specified otherwise, statistical analyses were performed in R (version 4.1.2; R Foundation for Statistical Computing, Vienna, Austria). Partitioning Around Medoids clustering based on Brays-Curtis distances of phage family relative abundances was performed using the “pam” function in the cluster package. The “fviz_nbclust” function in the factoextra package suggested four clusters. Principal coordinates analysis (PCoA) was performed using the “cmdscale” function and the Brays-Curtis distances of phage family abundances. Kruskal-Wallis test was performed to identify phage families with significantly different relative abundance across clusters. Differences in phage relative abundances between subjects with and without liver steatosis, and between subjects with and without diabetes, were assessed using the LefSe tool (55), with *P* < 0.05 and log10 LDA score >2 considered significant. Taxa detected in at least 5% of samples were included. Additional differential abundance analysis of taxa was performed with ANCOM v2.1 and using the abundance scores (56), where an FDR significance threshold of 0.2 was used for calculation of W statistics. W statistics greater than or equal to the 60th percentile of the W distribution were considered significant. Associations between the presence of each phage and liver steatosis/diabetes were further determined by Firth’s penalized maximum likelihood logistic regression. Using the “logistf” function of the “logistf” package, logistic regression was performed for each threshold, to obtain odds ratios adjusted for age, and gender (AOR), and 95% confidence intervals (CIs). Pairwise correlations between the relative abundances of phage and bacterial species were performed using Spearman’s correlation, using the “cor” and “cor.mtest” functions. For putative bacterial hosts, Fisher’s exact test was used to determine whether detection rates were different between groups, while the Mann-Whitney test was used to determine whether relative abundances were different between groups.

## Data Availability

The shotgun metagenomic sequencing data supporting the conclusions of this article, as well as age, gender, diabetes status, and liver parameters are available in the NCBI Sequence Read Archive (SRA) with the BioProject accession number PRJNA734860. A completed STORMS (Strengthening The Organizing and Reporting of Microbiome Studies) checklist (57) is available at https://doi.org/10.5281/zenodo.12708577.

## SUPPLEMENTAL MATERIAL

Figure S1 msystems.00434-24-s0001.docx Detection rate of liver steatosis-associated *Crassvirales* phages in non-steatotic versus steatotic subjects with presence of *Prevotella copri*.

Table S1 msystems.00434-24-s0002.docx Demographic and clinical parameters of the 340 study participants.

Table S2 msystems.00434-24-s0003.xlsx Phage taxa detected in at least 5% of the study participants.

Table S3 msystems.00434-24-s0004.docx Association of demographic and clinical parameters with family-level phageome clusters.

Table S4 msystems.00434-24-s0005.docx Known or inferred host ranges and lifestyles of diabetes- and steatosis-associated phages.

Table S5 msystems.00434-24-s0006.xlsx Crassvirales phage species identified using the VirMAP pipeline and detected in at least 5% of the study participants.

ASM does not own the copyrights to Supplemental Material that may be linked to, or accessed through, an article. The authors have granted ASM a non-exclusive, world-wide license to publish the Supplemental Material files. Please contact the corresponding author directly for reuse.
