# Correction for Kwan et al., “Gut phageome in Mexican Americans: a population at high risk for metabolic dysfunction-associated steatotic liver disease and diabetes”

**DOI:** 10.1128/msystems.01297-24

**Published:** 2024-10-18

**Authors:** Suet-Ying Kwan, Caroline M. Sabotta, Lorenzo R. Cruz, Matthew C. Wong, Nadim J. Ajami, Joseph B. McCormick, Susan P. Fisher-Hoch, Laura Beretta

## AUTHOR CORRECTION

Volume 9, no. 9, e00434-24, 2024, https://doi.org/10.1128/msystems.00434-24. Page 13, Acknowledgments, paragraph 2: The following should be added: “L.R.C. was supported by the 1R25CA240137 UPWARDS Training Program. L.R.C. was also supported by the CPRIT Research Training Award CPRIT Training Program (RP210028).”

