# Supplementary material for: Gut phageome in Mexican Americans: a population at high risk for metabolic dysfunction-associated steatotic liver disease and diabetes
Source: mSystems. 2024 Aug 21;9(9):e00434-24. doi: 10.1128/msystems.00434-24 (PMC11406975; doi:10.1128/msystems.00434-24)

**Supplementary Figure S1** Detection rate of liver steatosis-associated *Crassvirales* phages in non-steatotic versus steatotic subjects with presence of *Prevotella copri.*


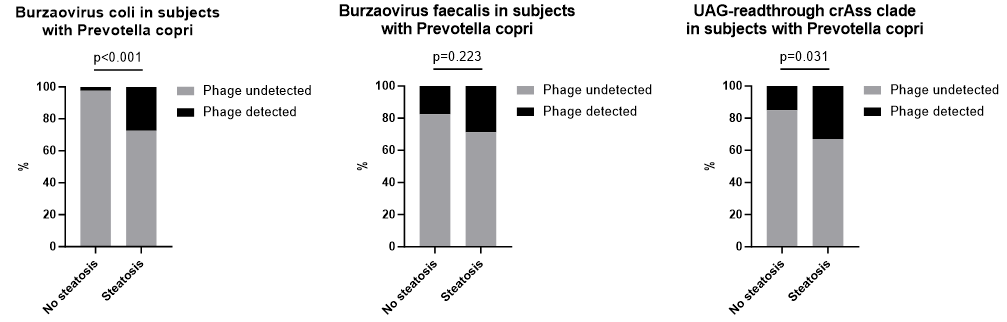

Supplement: Figure S1 — Detection rate of liver steatosis-associated Crassvirales phages in non-steatotic versus steatotic subjects with presence of Prevotella copri. [file msystems.00434-24-s0001.docx]
