# Supplementary material for: Gut phageome in Mexican Americans: a population at high risk for metabolic dysfunction-associated steatotic liver disease and diabetes
Source: mSystems. 2024 Aug 21;9(9):e00434-24. doi: 10.1128/msystems.00434-24 (PMC11406975; doi:10.1128/msystems.00434-24)
Supplement: Table S1 — Demographic and clinical parameters of the 340 study participants. [file msystems.00434-24-s0002.docx]

**Supplementary Table S1** Demographic and clinical parameters of the 340 study participants. BMI, body mass index; HbA1c, hemoglobin A1c; CAP, controlled attenuation parameter measured by FibroScan; LSM, liver stiffness measurement measured by FibroScan; AST, aspartate aminotransferase; ALT, alanine aminotransferase; HDL, high-density lipoprotein; LDL, low-density lipoprotein.

| **Parameter** | **Mean (range) - median or frequency (%)** |
| --- | --- |
| **Country of Birth (n=339)** |  |
| Mexico | 239 (70.5%) |
| USA | 94 (27.7%) |
| Other | 6 (1.8%) |
| **Age of arrival in Brownsville (n=340)** | 25.0 (0.0-69.0) - 26.0 |
| **Years in Brownsville (n=340)** | 30.1 (2.0-89.0) - 27.0 |
| **Male (n=340)** | 102 (30.0%) |
| **Age (n=340)** | 55.1 (18.0-89.0) - 57.0 |
| **BMI (n=339)** | 31.4 (16.7-50.0) - 30.8 |
| **Obese (n=339)** | 187 (55.2%) |
| **HbA1c (n=338)** | 6.6 (4.8-16.0) - 6.0 |
| **Diabetes (n=340)** | 124 (36.5%) |
| **Waist circumference (cm) (n=339)** | 104.1 (71.0-143.0) - 104.0 |
| **Waist-to-hip ratio (n=322)** | 0.9 (0.7-1.1) - 0.9 |
| **Hypertension (n=340)** | 118 (34.7%) |
| **Systolic blood pressure (n=339)** | 121.7 (87.0-202.0) - 119.0 |
| **Diastolic blood pressure (n=339)** | 72.5 (50.0-100.0) - 72.0 |
| **CAP (dB/m) (n=339)** | 290.7 (100.0-400.0) - 298.0 |
| **Liver Steatosis (CAP≥268) (n=339)** | 229 (67.6%) |
| **LSM (kPa) (n=340)** | 5.7 (1.9-45.5) - 4.6 |
| **Liver fibrosis status (n=340)** |  |
| No fibrosis (LSM <7.1 kPa) | 292 (85.9%) |
| Fibrosis (LSM ≥7.1 kPa) | 48 (14.1%) |
| Advanced fibrosis (LSM ≥8.8 kPa) | 29 (8.5%) |
| **Alcohol intake (g/day) (n=323)** | 3.4 (0.0-325.0) - 0.0 |
| **Drinking Status (n=323)** |  |
| Never | 213 (65.9%) |
| Moderate | 94 (29.1%) |
| Heavy | 16 (5.0%) |
| **Smoking Status (n=323)** |  |
| Never | 233 (72.1%) |
| Former | 67 (20.7%) |
| Current | 23 (7.1%) |
| **Blood Tests** |  |
| AST (U/L) (n=335) | 21.8 (6.0-205.0) - 19.0 |
| Abnormal AST (n=337) | 29 (8.6%) |
| ALT (U/L) (n=335) | 32.4 (12.0-173.0) - 27.0 |
| Abnormal ALT (n=337) | 114 (33.8%) |
| Albumin (gm/dL) (n=336) | 3.9 (3.0-4.6) - 3.9 |
| ALK (U/L) (n=336) | 90.1 (38.0-165.0) - 86.0 |
| Fasting blood glucose (n=321) | 114.1 (70.0-360.0) - 97.0 |
| Insulin (n=311) | 12.4 (0.6-85.9) - 10.6 |
| Triglycerides (n=338) | 153.0 (33.0-1596.0) - 126.0 |
| Total cholesterol (n=337) | 184.8 (50.0-318.0) - 185.0 |
| HDL cholesterol (n=338) | 51.6 (0.0-109.0) - 49.5 |
| LDL cholesterol (n=332) | 104.0 (8.0-204.0) - 105.0 |
| Platelets (x10^9^/L) (n=336) | 251.3 (116.0-480.0) - 247.0 |
