# Supplementary material for: Gut phageome in Mexican Americans: a population at high risk for metabolic dysfunction-associated steatotic liver disease and diabetes
Source: mSystems. 2024 Aug 21;9(9):e00434-24. doi: 10.1128/msystems.00434-24 (PMC11406975; doi:10.1128/msystems.00434-24)
Supplement: Table S3 — Association of demographic and clinical parameters with family-level phageome clusters. [file msystems.00434-24-s0004.docx]

**Supplementary Table 3** Association of demographic and clinical parameters with family-level phageome clusters. *p*-values were determined using binary logistic regression, comparing each cluster to the rest of the study population. Significant parameters and p-values (*p*<0.05) are in bold. BMI, body mass index; HbA1c, hemoglobin A1c; CAP, controlled attenuation parameter measured by VCTE-FibroScan; LSM, liver stiffness measurement as measured by VCTE-FibroScan; AST, aspartate aminotransferase; ALT, alanine aminotransferase; HDL, high-density lipoprotein; LDL, low-density lipoprotein. Data are presented as mean (range) – median or as frequency (%).

| **Variable** | **Cluster A (n=81)** | **p** | **Cluster B (n=149)** | **P** | **Cluster C (n=32)** | **p** | **Cluster D (n=78)** | **p** |
| --- | --- | --- | --- | --- | --- | --- | --- | --- |
| **Birth country (n=339)** |  |  |  |  |  |  |  |  |
| Mexico | 52 (64.2%) | Ref | 113 (75.8%) | Ref | 24 (75.0%) | Ref | 50 (64.9%) | Ref |
| USA | 28 (34.6%) | 0.124 | **32 (21.5%)** | **0.029** | 8 (25.0%) | 0.670 | 26 (33.8%) | 0.189 |
| Other | 1 (1.2%) | 0.766 | 4 (2.7%) | 0.360 | 0 (0.0%) | 0.988 | 1 (1.3%) | 0.801 |
| **Age of arrival in Brownsville (n=340)** | 22.2 (0.0-64.0) - 23.0 | 0.058 | **27.1 (0.0-68.0) - 29.0** | **0.029** | 26.0 (0.0-69.0) - 27.0 | 0.713 | 23.6 (0.0-67.0) - 24.5 | 0.355 |
| **Years in Brownsville (n=340)** | 30.4 (2.0-71.0) - 28.0 | 0.823 | 29.3 (6.0-89.0) - 25.0 | 0.442 | 31.7 (12.0-72.0) - 29.0 | 0.540 | 30.5 (5.0-74.0) - 27.5 | 0.800 |
| **Male (n=340)** | 24 (29.6%) | 0.934 | 45 (30.2%) | 0.943 | **15 (46.9%)** | **0.032** | 18 (23.1%) | 0.130 |
| **Age (n=340)** | 52.6 (19.0-76.0) - 57.0 | 0.071 | 56.5 (18.0-89.0) - 57.0 | 0.106 | 57.6 (39.0-87.0) - 57.0 | 0.293 | 53.9 (23.0-86.0) - 55.0 | 0.420 |
| **BMI (n=339)** | 31.6 (21.8-47.1) - 31.1 | 0.805 | 31.6 (16.7-50.0) - 30.6 | 0.689 | 31.9 (21.1-48.4) - 31.4 | 0.670 | 30.8 (18.5-49.0) - 30.1 | 0.308 |
| **Obese (n=339)** | 46 (56.8%) | 0.736 | 80 (53.7%) | 0.630 | 21 (65.6%) | 0.214 | 40 (51.9%) | 0.519 |
| **HbA1c (n=338)** | 6.6 (5.0-12.5) - 6.0 | 0.614 | 6.7 (4.9-14.1) - 6.1 | 0.352 | 6.2 (4.9-9.3) - 5.9 | 0.186 | 6.5 (4.8-16.0) - 5.9 | 0.497 |
| **Diabetes (n=340)** | 31 (38.3%) | 0.700 | **64 (43.0%)** | **0.029** | 7 (21.9%) | 0.077 | 22 (28.2%) | 0.086 |
| **Waist circumference (n=339)** | 104.6 (75.0-143.0) - 104.0 | 0.687 | 104.3 (76.0-141.0) - 104.0 | 0.745 | 105.7 (81.0-142.0) - 104.5 | 0.484 | 102.3 (71.0-131.0) - 103.0 | 0.200 |
| **Waist-to-hip ratio (n=322)** | 0.9 (0.7-1.1) - 1.0 | 0.669 | 1.0 (0.8-1.1) - 1.0 | 0.099 | 0.9 (0.8-1.0) - 0.9 | 0.705 | 0.9 (0.8-1.1) - 0.9 | 0.204 |
| **Hypertension (n=340)** | 31 (38.3%) | 0.440 | 54 (36.2%) | 0.599 | 12 (37.5%) | 0.727 | 21 (26.9%) | 0.102 |
| **Systolic blood pressure (n=339)** | 123.2 (92.0-191.0) - 118.0 | 0.400 | 122.5 (89.0-176.0) - 122.0 | 0.469 | 122.9 (87.0-171.0) - 120.0 | 0.690 | **118.0 (90.0-202.0) - 114.0** | **0.047** |
| **Diastolic blood pressure (n=339)** | 73.1 (50.0-93.0) - 73.0 | 0.546 | 72.7 (50.0-96.0) - 72.0 | 0.814 | 72.8 (51.0-89.0) - 73.0 | 0.840 | 71.6 (56.0-100.0) - 71.0 | 0.302 |
| **CAP (dB/m) (n=339)** | 294.2 (100.0-400.0) - 305.0 | 0.545 | 293.9 (118.0-400.0) - 298.0 | 0.374 | 299.7 (109.0-400.0) - 302.5 | 0.368 | **277.1 (144.0-400.0) - 284.5** | **0.023** |
| **Liver steatosis (CAP≥268) (n=339)** | 53 (66.2%) | 0.776 | 108 (72.5%) | 0.087 | 26 (81.2%) | 0.089 | **42 (53.8%)** | **0.004** |
| **LSM (kPa) (n=340)** | 5.5 (1.9-23.8) - 4.4 | 0.627 | 6.1 (2.4-45.5) - 4.7 | 0.170 | 5.8 (2.8-12.1) - 5.5 | 0.916 | 5.2 (2.2-20.8) - 4.4 | 0.221 |
| **Fibrosis (LSM ≥7.1 kPa) (n=340)** | 14 (17.3%) | 0.350 | 21 (14.1%) | 0.991 | 6 (18.8%) | 0.431 | 7 (9.0%) | 0.143 |
| **Advanced fibrosis (LSM ≥8.8 kPa) (n=340)** | 9 (11.1%) | 0.343 | 12 (8.1%) | 0.782 | 2 (6.2%) | 0.629 | 6 (7.7%) | 0.763 |
| **Alcohol intake (n=323)** | 3.2 (0.0-81.9) - 0.0 | 0.888 | 4.4 (0.0-325.0) - 0.0 | 0.464 | 2.3 (0.0-28.4) - 0.0 | 0.762 | 2.3 (0.0-23.7) - 0.0 | 0.590 |
| **Drinking status (n=323)** |  |  |  |  |  |  |  |  |
| Never | 51 (63.7%) | Ref | 97 (68.3%) | Ref | 17 (63.0%) | Ref | 48 (64.9%) | Ref |
| Moderate | 24 (30.0%) | 0.765 | 40 (28.2%) | 0.628 | 8 (29.6%) | 0.876 | 22 (29.7%) | 0.867 |
| Heavy | 5 (6.2%) | 0.514 | 5 (3.5%) | 0.273 | 2 (7.4%) | 0.531 | 4 (5.4%) | 0.821 |
| **Smoking status (n=323)** |  |  |  |  |  |  |  |  |
| Never | 55 (68.8%) | Ref | 104 (73.2%) | Ref | 19 (70.4%) | Ref | 55 (74.3%) | Ref |
| Former | 18 (22.5%) | 0.584 | 29 (20.4%) | 0.844 | 7 (25.9%) | 0.558 | 13 (17.6%) | 0.470 |
| Current | 7 (8.8%) | 0.468 | 9 (6.3%) | 0.613 | 1 (3.7%) | 0.524 | 6 (8.1%) | 0.790 |
| **AST (n=335)** | 22.1 (7.0-77.0) - 19.5 | 0.770 | 21.3 (8.0-57.0) - 20.0 | 0.551 | 28.2 (12.0-205.0) - 19.0 | 0.057 | 19.6 (6.0-64.0) - 18.5 | 0.088 |
| **Abnormal AST (n=337)** | 9 (11.1%) | 0.359 | 11 (7.4%) | 0.498 | **6 (18.8%)** | **0.038** | 3 (3.9%) | 0.113 |
| **ALT (n=335)** | 33.4 (14.0-128.0) - 26.5 | 0.597 | 32.1 (12.0-173.0) - 27.0 | 0.763 | 36.9 (13.0-100.0) - 32.0 | 0.142 | 30.3 (13.0-114.0) - 25.0 | 0.217 |
| **Abnormal ALT (n=337)** | 31 (38.3%) | 0.333 | 46 (31.1%) | 0.346 | 12 (37.5%) | 0.645 | 25 (32.9%) | 0.845 |
| **Albumin (n=336)** | 3.9 (3.5-4.5) - 4.0 | 0.207 | 3.9 (3.0-4.6) - 3.9 | 0.417 | 3.9 (3.3-4.3) - 4.0 | 0.554 | 3.9 (3.1-4.5) - 3.9 | 0.463 |
| **ALK (n=336)** | 87.5 (48.0-165.0) - 86.5 | 0.283 | **93.7 (43.0-159.0) - 88.0** | **0.022** | 84.5 (51.0-149.0) - 85.5 | 0.179 | 88.4 (38.0-157.0) - 84.0 | 0.486 |
| **Fasting blood glucose (n=321)** | 113.8 (74.0-262.0) - 95.0 | 0.943 | 118.6 (70.0-360.0) - 101.0 | 0.117 | 103.2 (77.0-195.0) - 95.0 | 0.202 | 109.8 (79.0-321.0) - 95.0 | 0.352 |
| **Insulin (n=311)** | 13.1 (0.6-85.9) - 10.9 | 0.399 | 12.5 (1.9-46.8) - 10.9 | 0.764 | 13.4 (3.3-35.0) - 9.6 | 0.558 | 11.0 (1.4-51.4) - 10.0 | 0.112 |
| **Triglycerides (n=338)** | 140.9 (50.0-436.0) - 112.0 | 0.307 | 155.9 (39.0-895.0) - 129.5 | 0.697 | 159.8 (33.0-368.0) - 135.5 | 0.741 | 157.4 (36.0-1596.0) - 121.0 | 0.722 |
| **Total cholesterol (n=337)** | 185.7 (77.0-303.0) - 183.0 | 0.802 | 183.5 (50.0-272.0) - 187.5 | 0.599 | 182.8 (90.0-260.0) - 179.0 | 0.753 | 187.1 (109.0-318.0) - 182.0 | 0.557 |
| **HDL cholesterol (n=338)** | 53.4 (27.0-84.0) - 51.0 | 0.154 | 50.3 (0.0-84.0) - 49.0 | 0.099 | 48.4 (30.0-94.0) - 45.5 | 0.143 | 53.6 (28.0-109.0) - 52.0 | 0.128 |
| **LDL cholesterol (n=332)** | 104.8 (8.0-199.0) - 106.5 | 0.814 | 102.9 (29.0-175.0) - 108.0 | 0.583 | 102.4 (26.0-163.0) - 103.0 | 0.772 | 106.1 (38.0-204.0) - 97.0 | 0.539 |
| **Platelets (n=336)** | 250.6 (144.0-377.0) - 245.0 | 0.909 | 247.4 (116.0-432.0) - 247.0 | 0.297 | 253.2 (175.0-378.0) - 249.0 | 0.848 | 258.7 (119.0-480.0) - 249.5 | 0.222 |
