# Supplementary material for: Gut phageome in Mexican Americans: a population at high risk for metabolic dysfunction-associated steatotic liver disease and diabetes
Source: mSystems. 2024 Aug 21;9(9):e00434-24. doi: 10.1128/msystems.00434-24 (PMC11406975; doi:10.1128/msystems.00434-24)
Supplement: Table S4 — Known or inferred host ranges and lifestyles of diabetes- and steatosis-associated phages. [file msystems.00434-24-s0005.docx]

**Supplementary Table S4** Known or inferred host ranges and lifestyles of diabetes- and steatosis-associated phages. Phage annotation data was extracted from the PhageScope bacteriophage database.

| **Association with disease** | | **Phage species name** | **Host range from PhageScope** | **Predicted phage lifestyle** | **Number of virulent factors** | **Virulent factor proteins** |
| --- | --- | --- | --- | --- | --- | --- |
| **Diabetes-associated phages** | | | |  |  |  |
|  | Positive | Streptococcus phage phiARI0746 | Streptococcus pneumoniae | Temperate | 0 |  |
|  | Positive | Shigella virus VASD | Shigella sonnei | Temperate | 2 | Shiga toxin 1 subunit A  Shiga toxin 1 subunit B |
|  | Positive | Escherichia virus phiV10 | Escherichia coli | Temperate | 0 |  |
|  | Positive | Escherichia phage TL-2011b | Escherichia coli | Temperate | 0 |  |
|  | Positive | Escherichia virus If1 | Escherichia coli | Virulent | 0 |  |
|  | Positive | Stx2-converting phage 1717 | Escherichia coli O157:H7 | Temperate | 3 | Shiga toxin 2 subunit A  Shiga toxin 2 subunit B  Type III secreted effector NIeG |
|  | Positive | Escherichia virus 933W | Escherichia coli | Temperate | 2 | Shiga toxin 2 subunit A  Shiga toxin 2 subunit B |
|  | Positive | Enterobacteria phage mEp460 | Escherichia coli | Temperate | 0 |  |
|  | Positive | Enterobacteria phage cdtI | Escherichia coli | Temperate | 5 | Putative small terminase subunit  Large terminase subunit  Prophage protein  Putative portal protein  Putative protease/scaffold protein |
|  | Positive | Shigella virus 7502Stx | Shigella sonnei | Temperate | 2 | Shiga toxin 1 subunit A  Shiga toxin 1 subunit B |
|  | Positive | Escherichia virus HK106 | Escherichia coli | Temperate | 0 |  |
|  | Positive | Enterobacteria phage IME10 | Escherichia coli | Temperate | 0 |  |
|  | Positive | Salmonella virus HK620 | Escherichia coli | Temperate | 0 |  |
|  | Positive | Shigella virus Sf6 | Shigella flexneri | Temperate | 0 |  |
|  | Negative | Ceduovirus_u_s | NA | NA | NA |  |
| **Steatosis-associated phages** | | | |  |  |  |
|  | Positive | Streptococcus phage IC1 | Streptococcus pneumoniae | Temperate | 1 | Lytic amidase |
|  | Positive | uncultured crAssphage | NA | NA | NA |  |
|  | Positive | Escherichia virus HK633 | Escherichia coli | Temperate | 0 |  |
|  | Positive | crAssphage cr7_1 | NA | Virulent | 0 |  |
|  | Positive | UAG-readthrough crAss clade | NA | NA | NA |  |
|  | Positive | crAss-like viruses | NA | NA | NA |  |
|  | Positive | crAssphage cr85_1 | NA | Virulent | 0 |  |
|  | Positive | crAssphage cr124_1 | NA | Virulent | 0 |  |
|  | Negative | Lactococcus phage r1t | Lactococcus | Temperate | 0 |  |
|  | Negative | Lactococcus phage BK5-T | Lactococcus lactis | Temperate | 0 |  |
|  | Negative | Enterobacterial phage mEp390 | Escherichia coli | Temperate | 0 |  |
|  | Negative | Escherichia virus 24B | Escherichia coli | Temperate | 0 |  |
